# Investigating distinct clinical features and constructing a nomogram model for survival probability in adults with cerebellar high-grade gliomas

**DOI:** 10.1186/s12885-024-12580-4

**Published:** 2024-07-13

**Authors:** Tao Chang, Rui Zhang, Jiahao Gan, Yuan Yang, Yanhui Liu, Yan Ju, Xiaodong Niu, Qing Mao

**Affiliations:** 1 Department of Neurosurgery and Neurosurgery Research Laboratory, West China Hospital, Sichuan University, Chengdu, China; 2 Clinical Medicine School, Traditional Chinese Medicine of Jiangxi University, Jiangxi, China; 3 Department of Neurosurgery, West China Hospital, Sichuan University, No. 37 Guo Xue Xiang, Chengdu, 610041 China

**Keywords:** Cerebellar, High-grade glioma, Prognosis, Nomogram

## Abstract

**Background:**

The clinical features of cerebellar high-grade gliomas (cHGGs) in adults have not been thoroughly explored. This large-scale, population-based study aimed to comprehensively outline these traits and construct a predictive model.

**Methods:**

Patient records diagnosed with gliomas were collected from various cohorts and analyzed to compare the features of cHGGs and supratentorial HGGs (sHGGs). Cox regression analyses were employed to identify prognostic factors for overall survival and to develop a nomogram for predicting survival probabilities in patients with cHGGs. Multiple machine learning methods were applied to evaluate the efficacy of the predictive model.

**Results:**

There were significant differences in prognosis, with SEER-cHGGs showing a median survival of 7.5 months and sHGGs 14.9 months (*p* < 0.001). Multivariate Cox regression analyses revealed that race, WHO grade, surgical procedures, radiotherapy, and chemotherapy were independent prognostic factors for cHGGs. Based on these factors, a nomogram was developed to predict 1-, 3-, and 5-year survival probabilities, with AUC of 0.860, 0.837, and 0.810, respectively. The model’s accuracy was validated by machine learning approaches, demonstrating consistent predictive effectiveness.

**Conclusions:**

Adult cHGGs are distinguished by distinctive clinical features different from those of sHGGs and are associated with an inferior prognosis. Based on these risk factors affecting cHGGs prognosis, the nomogram prediction model serves as a crucial tool for clinical decision-making in patient care.

**Supplementary Information:**

The online version contains supplementary material available at 10.1186/s12885-024-12580-4.

## Introduction

Cerebellar gliomas are a prominent subset of central nervous system malignancies in children, but they are significantly less prevalent in adults [1–4]. Primarily, most cerebellar gliomas in the pediatric population are low-grade gliomas, which are less aggressive tumors, such as pilocytic astrocytomas, accounting for nearly one-third of such tumors [5, 6]. Cerebellar high-grade gliomas (cHGGs) in adults, including primarily cerebellar glioblastomas and a minority of primary or anaplastic gliomas categorized as WHO grade III, are extremely rare. Research indicates that cerebellar glioblastoma accounts for only 0.24% to 4.1% of all primary glioblastomas, substantially less than their supratentorial counterparts [7, 8].

Current research efforts are primarily concentrated on uncovering prognostic factors that influence survival in supratentorial glioblastomas [9]. However, investigations into cHGGs are limited, primarily due to their rarity and the small number of cases available for in-depth analysis. The majority of previous research on cHGGs largely stems from case reports and small series, indicating a substantial gap in knowledge about the properties and prognosis of cHGGs [10].

Recent molecular studies have revealed unique biological behavior specific to cerebellar tumors, distinct from those observed in supratentorial HGGs (sHGGs) [4, 11]. While therapeutic regimens for cerebellar and supratentorial HGGs are currently available, discernible variations in treatment efficacy or prognosis exist [8, 11]. This highlights the pressing necessity of investigating distinct clinical presentations and developing a specialized clinical prediction model tailored for cHGGs.

As practical and instructive research on cHGGs, this investigation delved into the distinctive clinical features and inferior prognosis of cHGGs. Furthermore, we identified crucial factors impacting prognosis and devised a tailored predictive model for adult cHGGs.

## Methods

### Study population and data collection

This study collected clinical files for 512 cHGGs from the 18 registries of the SEER database (SEER*Stat software version 8.3.6) (S-cHGG), according to the International Classifcation of Diseases for Oncology, third edition (ICD-O-3). An additional cohort of 66 cHGGs was collected from the dataset of West China Hospital of Sichuan University (W-cHGG). For the sHGGs cohort, we accessed the clinical records of 606 cases from the TCGA database (https://portal.gdc.cancer.gov/) and 300 cases from the CGGA database (http://www.cgga.org.cn/) (Additional file 1: Figure S1).

The study enrolled participants aged ≥ 18 years with detailed clinical documentation and a definitive diagnosis of primary high-grade gliomas, including anaplastic oligodendroglioma (AO), anaplastic astrocytoma (AA), anaplastic oligoastrocytoma (AOA) and glioblastoma (GBM). Exclusion criteria were patients missing essential variables such as age, sex, survival duration, and prognosis. Patient records were collected during hospitalization, and follow-up information was obtained 3–6 months post-discharge via outpatient visits, telephone calls, and emails. This study was approved by our institutional review board of West China Hospital of Sichuan University (No. 2022.108).

### Variables and definitions

The patients included in the study were profiled according to different key variables, such as patient demographic files (age, gender, race), tumor characteristics (histopathology, WHO grade, tumor size), and treatment data (surgical resection, radiotherapy, and chemotherapy). Age was analyzed continuously and in categories, where the median value was used to divide patients into 18–57 years and ≥ 57 years. The year at diagnosis was defined as three intervals: 1973–2004, 2005–2009, and 2010–2016, highlighting the significant role of radiotherapy plus concomitant and adjuvant temozolomide in HGGs management since 2005. Tumor sizes were classified based on the median value into either < 38.0 mm or ≥ 38.0 mm. Tumor grades were divided into WHO grade III and IV, based on the 2016 World Health Organization classification of central nervous system tumors. For more details, see https://seer.cancer.gov/seerstat/variables/seer/brain_cns-recode/. For surgical management, the extent of resection was categorized as gross total resection (GTR), subtotal resection (STR), or partial resection (PR). Overall survival (OS) was defined as the period from surgery to patient death or the last follow-up.

### Cox analyses and nomogram construction

To pinpoint independent prognostic factors for OS, both univariable and multivariable Cox regression analyses were conducted. Variables such as race, WHO grade, surgery, chemotherapy, and radiotherapy were incorporated to construct a nomogram model. The effectiveness and credibility of the model were appraised by receiver operating characteristics, where values range from 0.5 to 1.0, and a higher value indicates superior discriminative capability. Additionally, calibration plots and decision curve analysis were utilized to verify the agreement between the predicted outcomes and the actual observations.

### Model evaluation and interpretation

To evaluate the model’s performance, multiple machine learning methods were employed using the “tidymodels” R package. Additionally, to evaluate the importance of variables in the nomogram, we measured SHAP values and various metrics, including an increase in mean squared error (IncMSE) and node purity (IncNodePurity). Additionally, SHAP interaction values were analyzed to illustrate how interactions between variables affect their impact on the predicted outcomes.

### Statistical analyses

Data analysis was conducted with R software (R Foundation, Vienna, Austria, version 4.1.2). The method of multiple imputation by chained equations (MICE) was adopted for missing values, excluding variables with more than 5% missing data from our analysis. We assessed categorical data using the Chi-square test or Fisher’s exact test. The log-rank test was applied to determine Kaplan-Meier survival curve differences. Both univariate and multivariate Cox regression analyses were performed to explore independent prognostic factors among the clinical variables. ANOVA was used to detect significant differences across multiple groups. If ANOVA showed significant results, Tukey’s honestly significant difference (Tukey HSD) test was used for post hoc analysis. The Wilcoxon rank sum test (also known as the Mann‒Whitney U test) was utilized to compare two independent samples. Dunn’s test was applied for nonparametric comparisons among multiple independent samples without normally distributed data.

Variables with a *p* value < 0.1 in univariate analysis were advanced to multivariate Cox regression analysis. The “forestplot” package in R was used to display the *p* value, hazard ratio, and 95% confidence interval of each variable. The ability of the nomogram to predict the prognosis of cHGG patients was evaluated by constructing receiver operating characteristic (ROC) curves in the “rms” R package. Further model interpretation, including the SHAP summary plot and interaction analysis, was carried out using the “xgboost” and “shapviz” R packages. The “ingredients” package helped determine variable importance, pinpointing the features most crucial to the model’s predictive accuracy. All the statistical tests were two-sided, with a *p* value < 0.05 indicating statistical significance.

## Results

### Clinical characteristics

A summary of the clinical features is shown in Table 1. Patients with cHGGs were older than those with sHGGs (S-cHGGs 56.5 ± 18.3, W-cHGGs 54.4 ± 17.3, and sHGGs 51.1 ± 14.0) (*p* < 0.001). In terms of demographics, a greater prevalence of HGG was observed in males and white individuals, regardless of tumor location. The median diameter of tumors in cHGGs was approximately 38.0 mm, with more than 55.0% of these tumors ≥ 38.0 mm, highlighting a significant risk of malignant mass effects. Regarding the pathological type, there was a greater incidence of GBM in infratentorial tumors, with 70.5% in the S-cHGGs cohort and 87.9% in the W-cHGGs group, compared to approximately 65.5% in the sHGGs cohort. Overall, 50.8% in S-cHGGs underwent gross total resection surgery. However, the application of radiotherapy and chemotherapy was significantly less common for cHGGs than for those with sHGGs. Moreover, the median survival time was considerably shorter for cHGGs, at only 7.5 months for S-cHGGs, as opposed to 14.9 months for sHGGs. The difference in postoperative adjuvant treatments, such as radiotherapy and chemotherapy, was statistically significant between supratentorial and infratentorial HGGs.

**Table 1:** Clinical features of high-grade gliomas in adults across three cohorts

	**S-cHGGs (*n* = 512)**	**W-cHGGs (*n* = 66)**	**sHGGs (*n* = 909)**	***P* value**
Year at diagnosis (%)				**< 0.001**
1973–2004	218 (42.6)	9 (13.6)	-	
2005–2009	91 (17.8)	23 (34.8)	-	
2010–2016	203 (39.6)	34 (51.6)	-	
Age (year)	56.5 ± 18.3	54.4 ± 17.3	51.1 ± 14.0	**< 0.001**
Age (%)				**< 0.001**
18–57 years	233 (45.5)	44 (66.7)	744 (81.8)	
≥ 57 years	279 (54.5)	22 (33.3)	165 (18.2)	
Gender (%)				0.862
Male	306 (59.8)	38 (57.6)	551 (60.6)	
Female	206 (40.2)	28 (42.4)	358 (39.4)	
Race (%)				**< 0.001**
White	475 (92.8)	0	545 (60.0)	
Black	37 (7.2)	0	64 (7.0)	
Asian	-	66 (100%)	300 (33.0)	
Tumor size (mm)	38 (30.0, 43.0)	38 (29.9, 43.8)		0.982
Tumor size (%)				1.000
< 38 mm	229 (44.7)	29 (43.9)	-	
≥ 38 mm	283 (55.3)	37 (56.1)	-	
Pathological type (%)				**< 0.001**
AO	9 (1.8)	2 (3.0)	88 (9.68)	
AA	142 (27.7)	6 (9.1)	167 (18.4)	
AOA	0	0	59 (6.5)	
GBM	361 (70.5)	58 (87.9)	595 (65.5)	
WHO grade (%)				**< 0.001**
Grade III	151 (29.5)	8 (12.1)	314 (34.5)	
Grade IV	361 (70.5)	58 (87.9)	595 (65.5)	
Surgery type (%)				0.702
PR/STR	252 (49.2)	29 (43.9)	-	
GTR	260 (50.8)	37 (56.1)	-	
Radiotherapy (%)				**< 0.001**
Yes	294 (57.4)	38 (57.6)	836 (92.0)	
No	218 (42.6)	28 (42.4)	73 (8.0)	
Chemotherapy (%)				**< 0.001**
Yes	248 (48.4)	37 (56.1)	646 (71.1)	
No	264 (51.6)	29 (43.9)	263 (28.9)	
Survival time (Month)	7.5 (2.0, 19.0)	8.0 (2.0, 25.0)	14.9 (8.3, 30.0)	**< 0.001**
Status on OS (%)				**< 0.001**
Dead	443 (86.5)	58 (87.9)	535 (58.9)	
Alive	69 (13.5)	8 (12.1)	374 (41.4)	
Therapy (%)				**0.015**
PR/STR	79 (15.4)	10 (15.6)	-	
PR/STR + Radio	49 (9.6)	2 (3.0)	-	
PR/STR + Chemo	24 (4.7)	8 (12.1)	-	
PR/STR + Radio + Chemo	100 (19.5)	9 (13.6)	-	
GTR	87 (17.0)	10 (15.6)	-	
GTR + Radio	49 (9.6)	7 (10.6)	-	
GTR + Chemo	28(5.5)	-	-	
GTR + Radio + Chemo	96 (18.8)	20 (30.3)	-	

The significant differences in diagnosis age, pathological type, and WHO grade for S-cHGGs and W-cHGGs underscored the heterogeneity in the biological behavior of cHGGs among distinct ethnicities (*p* < 0.05) (Additional file 2: Table S1).

### Survival time

Significant differences were observed in the survival durations of HGGs across various subgroups based on factors such as age, gender, pathological type, WHO grade, surgical procedure, and treatment modalities, including radiotherapy and chemotherapy (*p* < 0.05 for all). Specifically, within the S-cHGGs cohort, notable differences in survival were identified between subgroups differentiated by WHO grade, surgical procedure, radiotherapy, chemotherapy, only gross total resection (GTR) versus partial resection/subtotal resection (PR/STR), and GTR followed by chemoradiotherapy versus PR/STR combined with chemoradiotherapy (*p* < 0.05 for all) (Table 2). Additionally, the results presented pairwise comparisons of the survival time of adult HGGs in the three cohorts (Additional file 3: Table S2).

**Table 2:** The survival time of high-grade gliomas in adults across three cohorts

	**S-cHGGs (*n* = 512)**	**W-cHGGs (*n* = 66)**	**sHGGs (*n* = 909)**	***P* value**
Age (%)				
18–57 years	8.0 (3.0, 21.0)	9.5 (2.5, 26.5)	18.4 (9.7, 46.6)	**< 0.001**
≥ 57 years	7.0 (2.0,18.0)	5.7 (2.1, 19.2)	11.8 (7.3, 18.7)	**< 0.001**
Gender (%)				
Male	7.0 (2.0, 18.8)	11.0 (2.3, 24.5)	15.0 (8.5, 27.0)	**< 0.001**
Female	8.0 (3.0,19.0)	6.5 (2.0, 27.2)	14.9 (8.0, 36.7)	**< 0.001**
Race (%)				
White	8.0 (2.0, 20.0)	-	-	-
Black	7.0 (2.0, 13.0)	-	-	-
Tumor size (%)				
< 38 mm	7.0 (2.0, 18.0)	9.0 (3.0, 27.0)	-	0.293
≥ 38 mm	8.0 (2.0, 20.0)	7.0 (2.0, 25.0)	-	0.900
Pathological type (%)				
AO	14.0 (5.0, 22.0)	9.5 (5.3, 13.8)	38.4 (14.2, 71.8)	0.061
AA	9.0 (3.0, 18.8)	19.0 (2.0, 84.0)	21.6 (11.2, 51.4)	**< 0.001**
GBM	7.0 (2.0, 19.0)	8.0 (2.3, 24.5)	12.6 (7.6, 21.0)	**< 0.001**
WHO grade (%)^§^				
Grade III	9.0 (3.0, 19.0)	10.0 (1.8, 52.0)	24.5 (11.6, 62.1)	**< 0.001**
Grade IV	7.0 (2.0, 19.0)	8.0 (2.3, 24.5)	12.6 (7.6, 21.0)	**< 0.001**
Surgery type (%)^&^				
GTR	9.0 (3.0, 25.0)	17.0 (4.8, 47.5)	-	**0.003**
PR/STR	7.0 (2.0, 15.2)	8.0 (2.0, 25.0)	-	0.659
Radiotherapy (%)^#^				
Yes	11.0 (6.0, 23.8)	16.0 (6.3, 33.0)	32.1 (10.0, 66.8)	**< 0.001**
No	3.0 (1.0, 9.0)	2.0 (1.0, 8.2)	14.9 (8.4, 28.5)	**< 0.001**
Chemotherapy (%)^∮^				
Yes	13.0 (6.0, 25.0)	16.0 (6.0, 34.0)	14.3 (8.2, 29.1)	0.054
No	3.0 (1.0, 11.2)	2.0 (1.0, 13.0)	16.1 (9.2, 31.1)	**< 0.001**
Status on OS (%)^£^				
Dead	7.0 (2.0, 16.0)	6.5 (2.0, 18.0)	13.7 (7.4, 49.3)	**< 0.001**
Alive	20.0 (7.0, 68.0)	18.5 (5.8, 76.2)	15.3 (9.5, 25.9)	**< 0.001**
Therapy (%)				
GTR^⁋^	2.0 (1.0, 10.0)	4.0 (2.0, 9.0)	-	0.124
GTR + Radio	10.0 (4.0, 18.0)	12.5 (6.8, 14.2)	-	0.392
GTR + Chemo	10.0 (5.0, 24.2)	11.5 (8.2, 22.5)	-	0.065
GTR + Radio + Chemo^∬^	18.5 (6.0, 33.0)	18.0 (16.0, 70.0)	-	**0.032**
PR/STR	1.0 (1.0, 4.5)	1.0 (1.0, 2.8)	-	0.191
PR/STR + Radio	8.0 (3.0, 14.0)	10.5 (6.8, 14.2)	-	0.789
PR/STR + Chemo	7.0 (4.0, 17.2)	-	-	-
PR/STR + Radio + Chemo	10.0 (7.0, 21)	10.5 (6.0, 24.0)	-	0.166

There were significant difference in survival time among various subgroups by WHO grade (*p* = 0.028)^§^, surgical procedures (*p* = 0.003)^&^, radiotherapy (*p* < 0.001)^#^, chemotherapy (*p* < 0.001)^∮^, status (*p* < 0.001)^£^, only GTR versus PR/STR procedure (*p* = 0.032)^⁋^, GTR + Radio + Chemo versus PR/STR + Radio + Chemo therapy (*p* = 0.014)^∬^ in S-cHCGs cohort

### Univariate and multivariate Cox regression analyses

**Fig. 1:**
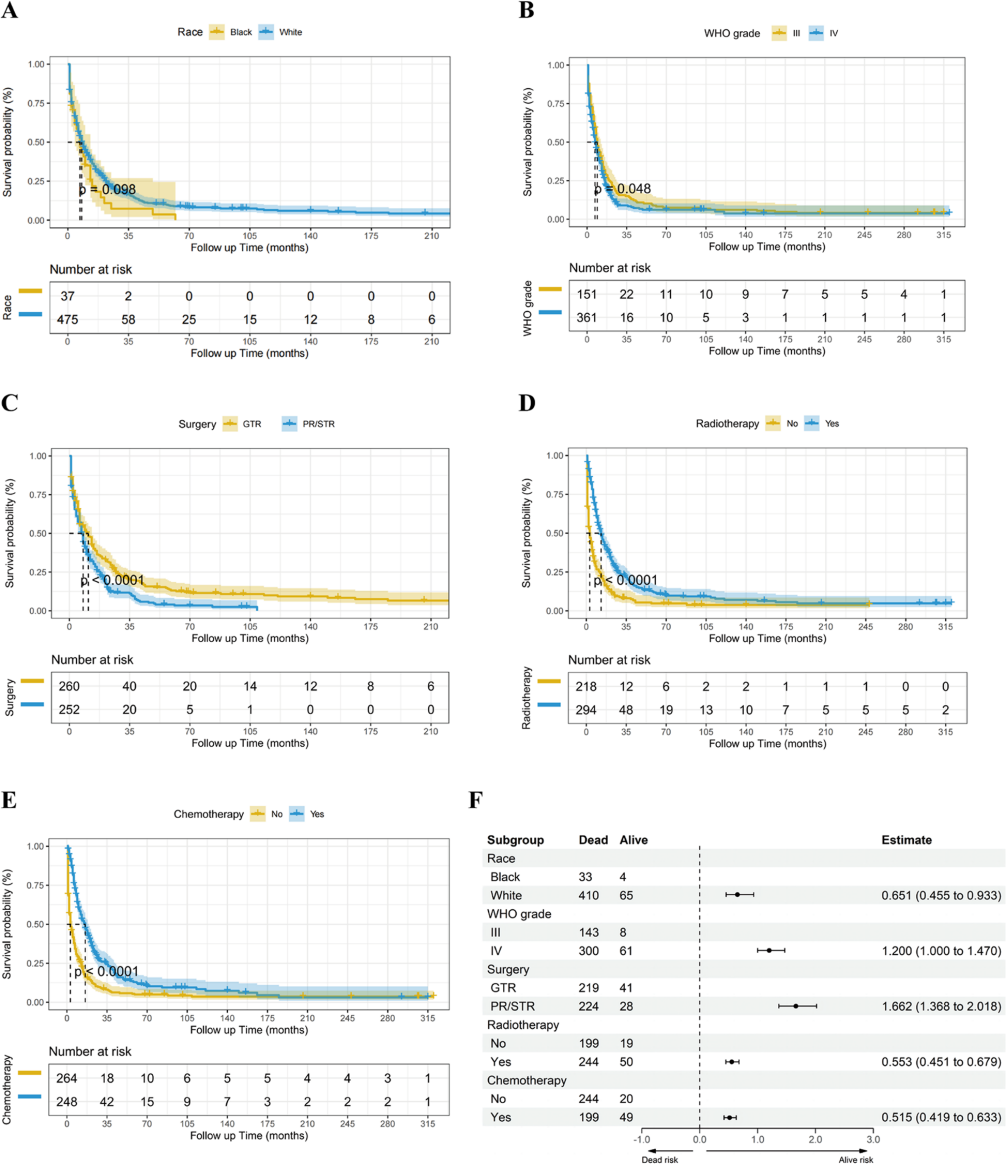
Variables affecting the overall survival of patients with cerebellar high-grade gliomas (cHGGs) in the SEER database. Kaplan-Meier survival curves stratified by race (*p* < 0.1) (**A**), WHO grade (*p* < 0.05) (**B**), surgery (*p* < 0.001) (**C**), radiotherapy (*p* < 0.001) (**D**), and chemotherapy (*p* < 0.001) (**E**). (**F**) Forest plot assessing the associations between these five variables and prognosis in patients with cHGGs

Variables such as race, WHO grade, type of surgery, radiotherapy, and chemotherapy, with *p* values ≤ 0.1, were identified as potentially significant and were thus selected for further analysis (Table 3). Further exploration using Kaplan-Meier analysis assessed the influence of these variables on overall survival. These findings indicated that white patients with WHO grade III, maximal resection, radiotherapy, and chemotherapy experienced improved survival outcomes than their counterparts (Fig. 1A-E). Race (HR 0.651, 95% CI 0.455–0.933, *p* = 0.019), WHO grade (HR 1.200, 95% CI 1.000–1.470, *p* < 0.01), surgery procedures (HR 1.662, 95% CI 1.368–2.018, *p* < 0.001), radiotherapy (HR 0.553, 95% CI 0.451–0.679, *p* < 0.001), and chemotherapy (HR 0.515, 95% CI 0.419–0.633, *p* < 0.001) were independently confirmed as prognostic indicators of overall survival (Fig. 1F, Table 3). These findings emphasize the critical significance of these variables in predicting the prognosis of patients with cHGGs.

**Table 3:** Univariate and multivariate cox regression analysis in SEER-cHCGs cohort

	**Univariate**			**Multivariate**		
	**HR**	**(95% CI)**	***p* value**	**HR**	**(95% CI)**	***p* value**
Year 1973–2005 (preference)						
2005–2009	1.092	0.844–1.414	0.501			
2010–2016	1.144	0.926–1.414	0.212			
Age 18–57 / ≥ 57(years)	1.044	0.866–1.259	0.649			
Gender Male / Female	0.975	0.806–1.179	0.795			
Race Black / White	0.744	0.521–1.062	**0.098**	0.651	0.455–0.933	**0.019**
Tumor size < 3.8 / ≥ 3.8(cm)	0.899	0.745–1.085	0.266			
Pathylogical AO (preference)						
AA	1.351	0.662–2.759	0.408			
GBM	1.374	0.680–2.778	0.376			
WHO grade III / IV	1.037	0.848–1.267	**0.048**	1.200	1.000–1.470	**< 0.01**
Surgery GTR / PR/STR	1.464	1.210–1.772	**< 0.001**	1.662	1.368–2.018	**< 0.001**
Radiotherapy No / Yes	0.472	0.391–0.570	**< 0.001**	0.553	0.451–0.679	**< 0.001**
Chemotherapy No / Yes	0.464	0.384–0.560	**< 0.001**	0.515	0.419–0.633	**< 0.001**

**Fig. 2:**
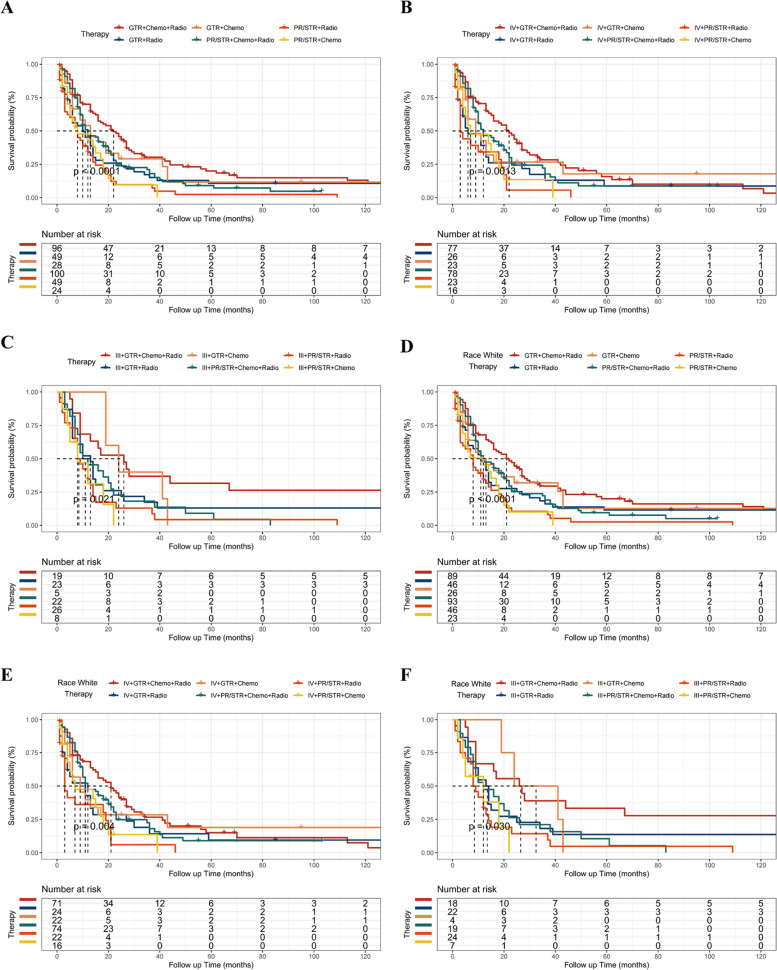
Effects of variable subgroups on the overall survival in the SEER database. Kaplan-Meier survival curves stratified by therapy (*p* < 0.001) (**A**), WHO grade IV (*p* < 0.01) (**B**), WHO grade III (*p* < 0.05) (**C**), white race (*p* < 0.001) (**D**), white race with WHO grade IV (*p* < 0.01) (**E**), and white race with WHO grade III (*p* < 0.05)

Patients with cHGGs, who received adjuvant radiotherapy and chemotherapy post-GTR, showed better outcomes, both in overall KM analysis (Fig. 2A) and in subtype analysis based on WHO grade (Fig. 2B and C) and racial background (Fig. 2D and E). However, for white patients with WHO grade III, GTR followed by chemoradiotherapy was associated with a better prognosis (Fig. 2F). These findings underscore the effectiveness of comprehensive treatment approaches in enhancing outcomes for cHGGs.

### Construction and evaluation of nomograms

**Fig. 3:**
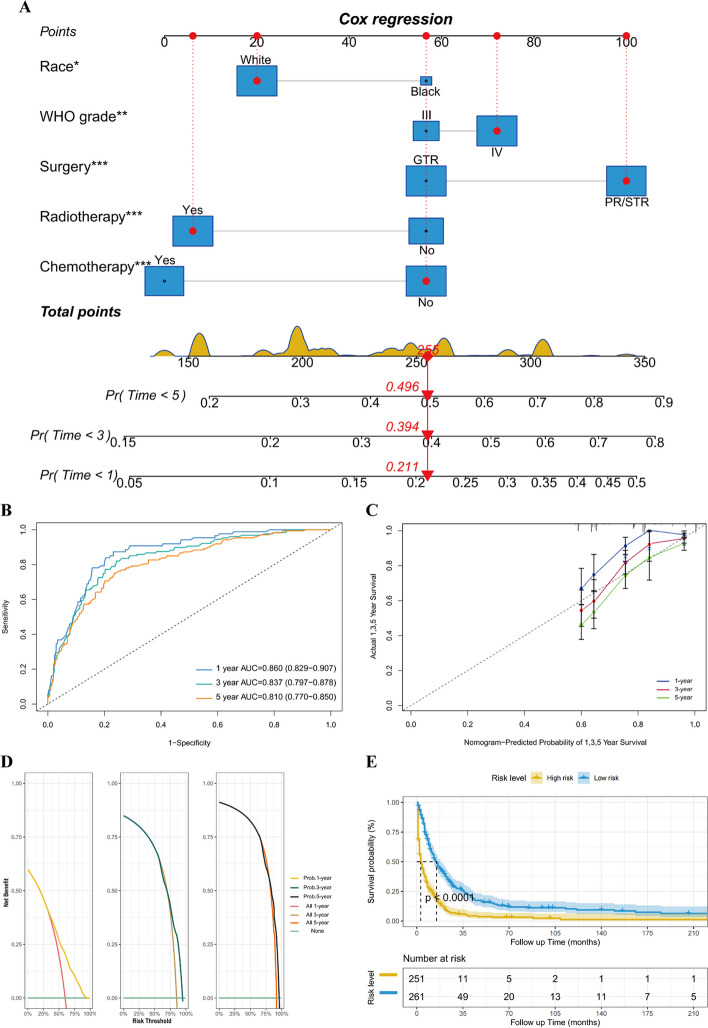
Model construction and validation for 1-, 3-, and 5-year overall survival (OS). **A** Nomogram model for OS. **B** Receiver operating characteristic curve for OS. Calibration plots (**C**) and decision curve analysis (**D**) of the 1-, 3-, and 5-year OS probabilities showing good consistency in cHGGs. **E** Kaplan-Meier curves for OS among risk-stratified subgroups of cHGGs (*p* < 0.001)

A nomogram was designed to predict the 1-, 3-, and 5-year probabilities of OS according to five critical variables, as illustrated in Fig. 3A. The predictive performance of the nomogram, as evidenced by the area under the curve (AUC) for 1, 3, and 5 years, reached 0.860 (95% CI 0.829–0.907), 0.837 (95% CI 0.797–0.878), and 0.810 (95% CI 0.770–0.850), respectively. The calibration curve and DCA for the 1-, 3- and 5-year OS probability indicated a noteworthy consistency between the predicted outcomes of the nomogram and the actual observations (Fig. 3B-D). Moreover, Kaplan‒Meier survival analysis, stratified by the model’s overall risk score, revealed that individuals with lower risk scores achieved significantly superior survival than their higher-risk counterparts, as illustrated in Fig. 3E.

### Evaluation of the performance of the prediction model

**Fig. 4:**
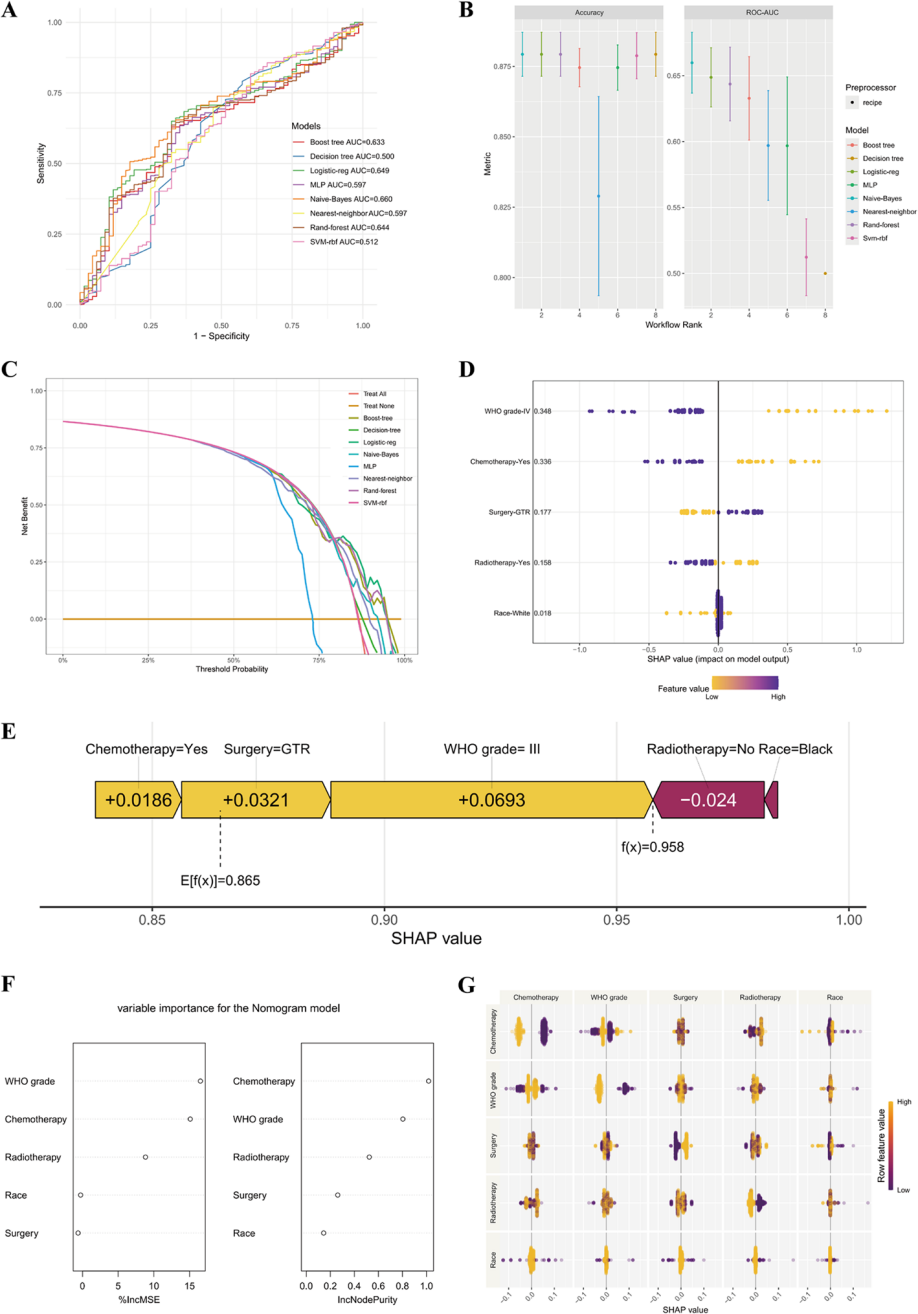
Machine learning approaches for the prognostic model. Time-dependent ROC curves (**A**) and accuracy (**B**) of the machine learning methods used for the prognostic model. **C** Decision curve for the prognostic model. **D** The SHAP summary plot revealed a diverse distribution of points, indicating that WHO grade and chemotherapy substantially impacted the model’s performance. **E** The SHAP value of each variable in the 306th patient. **F** IncMSE (panel left) and IncNodePurity (panel right). They also emphasized the notable influence of WHO grade and chemotherapy on the model’s predictive accuracy. **G** SHAP interactions among variables

Multiple machine-learning approaches were used to validate the comprehensive performance of the model prediction, which exhibited relatively strong power. Among these methods, the Nave-Bayes method presented the optimal predictive ability (AUC = 0.660), and the Decision tree method had the weakest predictive performance (AUC = 0.500) (Fig. 4A-C).

Moreover, five crucial variables significantly contributing to the predictive effectiveness were identified based on their high mean absolute SHAP scores. The SHAP summary plot revealed a diverse distribution of points, indicating that WHO grade and chemotherapy exerted substantial impacts on the model’s performance (Fig. 4D). For instance, the SHAP value for the 306th patient was 0.865 (displayed in the top right corner), illustrating that WHO grade III significantly increased the model’s predictive confidence, whereas the absence of radiotherapy diminished it (Fig. 4E). A lower %IncMSE value signifies a negligible effect on model performance, whereas a higher value indicates a notable enhancement. Compared with its absence, IncNodePurity measures the improvement in node purity when a feature is introduced, further emphasizing the critical roles of WHO grade and chemotherapy in enhancing the model’s predictive capability (Fig. 4F). Furthermore, SHAP interaction analysis revealed that race had a pronounced interaction effect with other variables (Fig. 4G).

## Discussion

Researches on the prognosis of patients with cHGGs have yielded varied conclusions for both pediatric and adult populations [7, 10–14]. Individuals with gliomas in the cerebellar region revealed an unfavorable prognosis compared to those in other regions for pediatric HGGs or adult GBM [15, 16]. In contrast, Babu et al. [17] reported that there was no significant difference in prognosis between individuals with cerebellar GBM and those with supratentorial GBM (7 months versus 8 months,* p* = 0.24). The median survival time for patients with either cGBM or sGBM was 8 months, but those with cGBM experienced notably greater survival at two and three years—21.5% and 12.7%, respectively—than the 8.0% and 5.3% for sGBM sufferers [14]. Contrasting with previous findings, we discovered that cHGGs typically resulted in less favorable outcomes than their supratentorial counterparts, with a median survival time of 7.5 years compared to 14.9 years. The poor prognosis of patients with cHGGs could be related to the tumor spreading into the brainstem and the diversity of glioblastomas [15]. This disparity indicates that cHGGs may represent a potentially more aggressive disease with distinct biological behaviors, presenting more complex treatment challenges and distinguishing it as a separate entity from sHGGs.

Current investigations into the impact of race on the prognosis for HGGs have shown variability. Specific analyses revealed better survival outcomes for Asians or Pacific Islanders than white and black populations across all evaluated timeframes [18, 19]. Alternative studies indicated improved survival rates for white patients, with notable comparisons showing a median survival of 13.0 months for Hispanic patients versus 24.3 months for white patients [19–21]. An underlying explanation might be that being Caucasian is independently associated with a better prognosis for patients receiving the Stupp regimen [22]. This study revealed that the median survival times for patients with cHGGs were 8.0 months for whites, 7.0 months for blacks, and 8.0 months for Chinese, indicating no significant survival disparities across these races. We posited that disparities in healthcare access, socioeconomic conditions, and comorbidities collectively affect survival in HGGs. Notably, race was an independent prognostic factor in multivariate analysis, albeit with a relatively modest effect, as indicated by the SHAP plot (Fig. 4D, F, G). This pointed to the possibility of intricate interactions between race and other variables affecting clinical outcomes across various racial groups.

Glioma grade has been recognized as a vital prognostic factor for survival, with a higher grade associated with worse outcomes [23, 24]. This conclusion is consistent with research on cHGGs, where WHO grade has been recognized as an independent prognostic risk factor [25]. The median survival time for patients with WHO grade III cHGGs was 9.0 months, and for those with WHO grade IV, it was only 7.0 months. This was significantly lower compared to patients with sHGGs, who had respective median survival times of 24.5 months and 12.6 months for WHO grades III and IV.

Current therapeutic strategies for managing cHGGs primarily revolve around surgery, radiotherapy, and chemotherapy. However, an optimal treatment protocol has yet to be established. Previous studies have highlighted the importance of maximal surgical resection for improving the prognosis of glioma patients [26]. Aligning with previous research on sHGGs [27–29], our research identified surgical procedures, particularly gross total resection, as an independent indicator of improved survival in patients with cHGGs. Moreover, the combination of maximal surgical resection with chemoradiotherapy markedly improved survival, with a median survival time of 18.5 months, while outcomes from solely using GTR or PR/STR were less favorable (2.0 months and 1.0 months, respectively). Compared to surgical procedures alone, supplementing surgical intervention with either radiotherapy or chemotherapy can also significantly increase survival. Regrettably, in both the S-cHGGs and W-cHGGs cohorts, approximately 32% of individuals did not receive adjuvant radiotherapy or chemotherapy following surgery.

The limited number of cases has prevented the establishment of predictive models based on the clinical features of cHGGs in previous literature [7, 14]. Here, a nomogram model incorporating five key variables, race, WHO grade, surgery, radiotherapy, and chemotherapy, exhibited impressive predictive performance [30]. The effectiveness and reliability of the nomogram model were validated by machine learning approaches, indicating that it could be a practical and intuitive tool for predicting the prognosis of individual cHGGs.

This retrospective study faces limitations arising from its dependence on a specific cohort from public databases. The exclusion of cases due to missing information might introduce selection bias, and there is variability in the definition of factors across cohorts. The demographic distribution in this study, with a heavy predominance of whites (92.5%) and a small percentage of black participants (7.2%), may limit the generalizability of these findings, highlighting the importance of demographic diversity in research outcomes. Molecular markers such as IDH1/2, 1p/19q, and MGMT, which are crucial for assessing prognosis, were unavailable in the SEER database. Additionally, the diversity of WHO tumor grades, indicative of molecular heterogeneity and a wide range of subclassifications, presented significant variability even within the same grade. The lack of imaging details on the extent of tumor invasion into the brainstem represented a critical omission that could influence patient prognoses. Despite these limitations, the findings of this study persist as scientifically sound and provide valuable guidance for the clinical management of cHGGs.

## Conclusions

Adult cHGGs have distinct clinical features that diverge from those of sHGGs and are associated with an inferior prognosis. The nomogram prediction model, based on crucial indicators affecting cHGGs prognosis, is a valuable tool for guiding clinical decision-making in patient care.

### Supplementary Information

Additional file 1: Figure S1. The flowchart for the selection of high-grade gliomas from the various databases. (A) SEER database. (B) TCGA database. (C) CGGA database.

Additional file 2: Table S1. Comparative analysis of clinical features for adults with high-grade gliomas among three cohorts.

Additional file 3: Table S2. Comparative analysis of survival time for adults with high-grade gliomas among three cohorts.

## Data Availability

The datasets used in this study are available in the SEER database (https://seer.cancer.gov/), TCGA database (https://portal.gdc.cancer.gov/) and CGGA database (http://www.cgga.org.cn/). The data from the West China Hospital of Sichuan University are available upon reasonable request.

## Abbreviations

cHGGs: Cerebellar high-grade gliomas
sHGGs: Supratentorial high-grade gliomas
S-cHGGs: SEER cerebellar high-grade gliomas
W-cHGGs: West China Hospital cerebellar high-grade gliomas
AA: Anaplastic astrocytoma
AO: Anaplastic oligodendroglioma
GBM: Glioblastoma
GTR: Gross total resection
STR: Subtotal resection
PR: Partial resection
OS: Overall survival
Radio: Radiotherapy
Chemo: Chemotherapy
IncMSE: Increase in mean squared error
IncNodePurity: Increase in node purity
MICE: Multiple imputation by chained equations
ROC: Receiver operating characteristic
