# Supplementary material for: Investigating distinct clinical features and constructing a nomogram model for survival probability in adults with cerebellar high-grade gliomas
Source: BMC Cancer. 2024 Jul 13;24:836. doi: 10.1186/s12885-024-12580-4 (PMC11245792; doi:10.1186/s12885-024-12580-4)
Supplement: Supplementary file 1 — Additional file 1: Figure S1. The flowchart for the selection of high-grade gliomas from the various databases. (A) SEER database. (B) TCGA database. (C) CGGA database. [file 12885_2024_12580_MOESM1_ESM.docx]

**
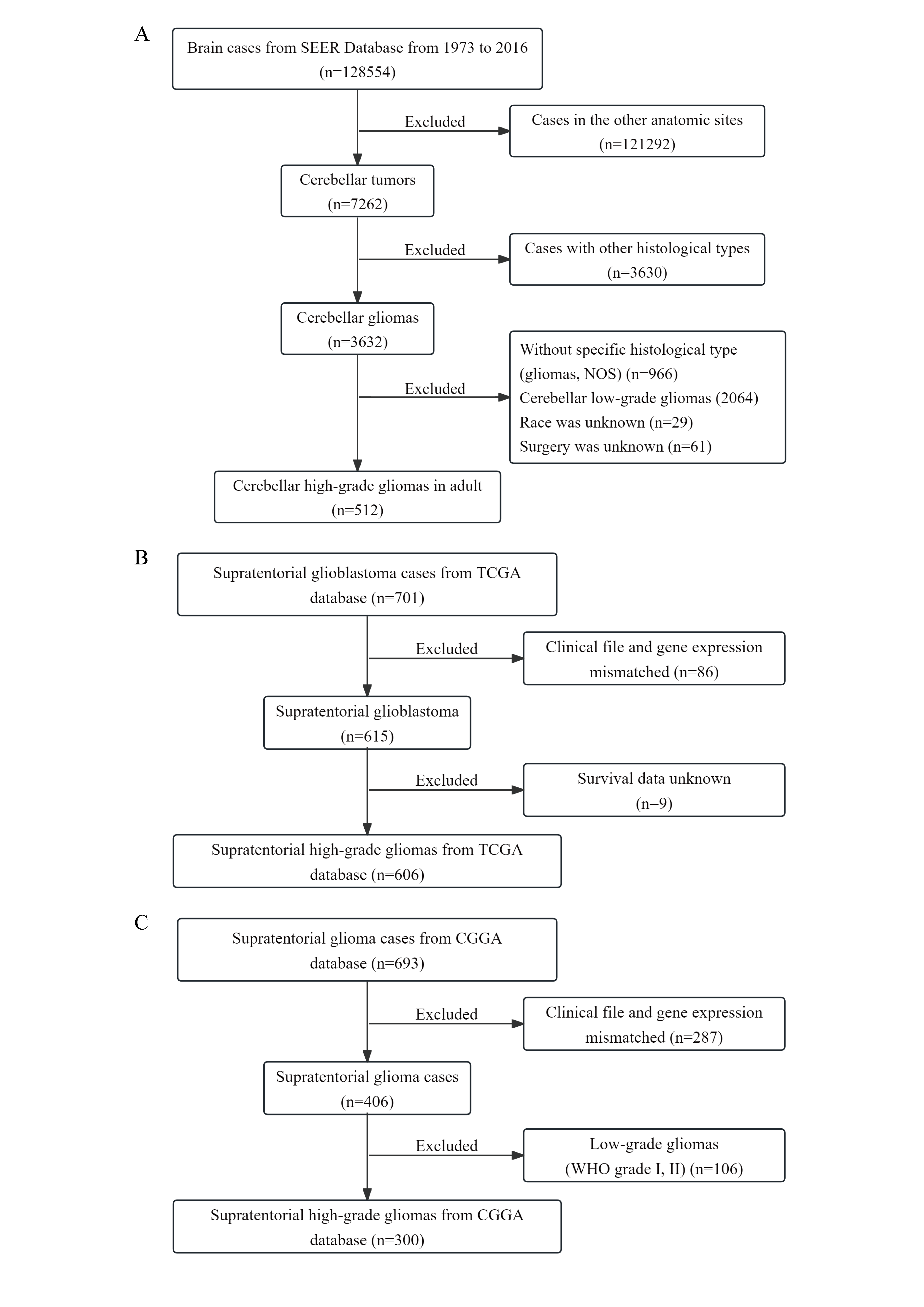
**

### **[Figure S1.](https://static-content.springer.com/esm/art:10.1186/s12885-024-12159-z/MediaObjects/12885_2024_12159_MOESM2_ESM.jpg)** The flowchart for the selection of high-grade gliomas from the various databases. (**A**) SEER database. (**B**) TCGA database. (**C**) CGGA database.
