# Supplementary material for: Investigating distinct clinical features and constructing a nomogram model for survival probability in adults with cerebellar high-grade gliomas
Source: BMC Cancer. 2024 Jul 13;24:836. doi: 10.1186/s12885-024-12580-4 (PMC11245792; doi:10.1186/s12885-024-12580-4)
Supplement: Supplementary file 2 — Additional file 2: Table S1. Comparative analysis of clinical features for adults with high-grade gliomas among three cohorts. [file 12885_2024_12580_MOESM2_ESM.docx]

**Table S1** Comparative analysis of clinical features for adults with high-grade gliomas among three cohorts

| **Variable** | **S-cHGGs vs. W-cHGGs** | | **S-cHGGs vs. sHGGs** | |
| --- | --- | --- | --- | --- |
|  | **χ^2^ / Z value** | ***p* value** | **χ^2^ / Z value** | ***p* value** |
| Age (year) | 0.851 | 0.197 | -3.933 | **<0.001** |
| Age (%) | 9.657 | **0.002** | 19.97 | **<0.001** |
| Gender (%) | 0.043 | 0.835 | 0.067 | 0.796 |
| Pathological type (%) | 10.867 | **0.004** | 42.369 | **<0.001** |
| WHO grade (%) | 7.997 | **0.004** | 3.570 | 0.058 |
| Radiotherapy (%) | 1.527 | 1.000 | 23.79 | **<0.001** |
| Chemotherapy (%) | 1.071 | 0.301 | 7.092 | **<0.001** |
| Survival time (Month) | 0.850 | 0.592 | -11.088 | **<0.001** |
| Status on OS (%) | 0.012 | 1.000 | 1.156 | **<0.001** |
