# Supplementary material for: Investigating distinct clinical features and constructing a nomogram model for survival probability in adults with cerebellar high-grade gliomas
Source: BMC Cancer. 2024 Jul 13;24:836. doi: 10.1186/s12885-024-12580-4 (PMC11245792; doi:10.1186/s12885-024-12580-4)
Supplement: Supplementary file 3 — Additional file 3: Table S2. Comparative analysis of survival time for adults with high-grade gliomas among three cohorts. [file 12885_2024_12580_MOESM3_ESM.docx]

| **Variable** | **S-cHGGs vs. W-cHGGs** | | **S-cHGGs vs. sHGGs** | |
| --- | --- | --- | --- | --- |
|  | **Z value *p* value** | | **Z value *p* value** | |
| Age (%) |  |  |  |  |
| 18-57 years | -0.926 | 0.180 | -8.870 | **<0.001** |
| ≥57 years | -0.113 | 0.455 | -5.727 | **<0.001** |
| Gender (%) |  |  |  |  |
| Male | -1.170 | 0.121 | -8.834 | **<0.001** |
| Female | 0.061 | 0.475 | -6.692 | **<0.001** |
| Pathological type (%) |  |  |  |  |
| AO | 0.605 | 0.273 | -1.840 | **0.033** |
| AA | -0.712 | 0.238 | -6.517 | **<0.001** |
| GBM | -1.135 | 0.128 | -7.964 | **<0.001** |
| WHO grade (%) |  |  |  |  |
| Grade III | -0.549 | 0.291 | -7.687 | **<0.001** |
| Grade IV | -1.135 | 0.128 | -7.964 | **<0.001** |
| Radiotherapy (%) |  |  |  |  |
| Yes | -1.220 | 0.167 | -4.274 | **<0.001** |
| No | 0.364 | 0.358 | -7.136 | **<0.001** |
| Chemotherapy (%) |  |  |  |  |
| Yes | -0.396 | 0.346 | -2.339 | **0.025** |
| No | 0.039 | 0.485 | -11.693 | **<0.001** |
| Status on OS |  |  |  |  |
| Dead | -0.303 | 0.381 | -11.854 | **<0.001** |
| Alive | -2.058 | **0.030** | 2.610 | **0.016** |

**Table S2** Comparative analysis of survival time for adults with high-grade gliomas among three cohorts
